# A Year's Work in the Hospitals and Medical Charities of London

**Published:** 1888-06-02

**Authors:** 


					June 2, 1888. THE HOSPITAL. 153
A Years Work in the Hospitals and Medical Charities of London.
The following Table and Figures should be studied witli the remarks following the Summary on the last page,
to make their bearing more intelligible and better understood.
NEWINGTON AND SOUTH DISTRICT.
Comprising Battersea, Wandsworth, Tooting, Balham, Streatham, Brixton, Lambeth, Ne wing ton, Southwark,
Bermondsey, Camberwell, Greenwich, Deptford, Lewisham, Blackheath, Woolwich, etc.
Special Note.?It will be seen that out of 1,586 available beds only 933 were daily occupied in this district during 1887. That is to say,
more than ose-third of the whole hospital accommodation was not available for the relief of the poor of the district for want of funds. As it is,
the total income was upwards of ?3,000 less than the expenditure entailed by the maintenance of the 933 beds actually occupied throughout tue
entire year. It would therefore appear that no additional hospital accommodation is needed, but something like ?35,000 per annum more money,
to maintain and open the 650 beds kept empty during 1887 in this district alone for want of funds.
No. of
Beds
Daily
Occu-
pied.
Hospitals.
410 Guy's
19 Miller ...
190 Seamen's
55 Evelina, for Children
38 Home for Sick Children
16 General Lying-in
47 Royal, for Children and Women
9 Royal South London Ophthalmic
2 Hospital for Diseases of the Skin
147 Metropolitan Convalescent ...
933
933
Dispensaries.
Battersea
Brixton, &c.
Camberwell Provident
Clapham
Forest Hill
Gipsy Hill
Royal South London...
South Lambeth
South London Medical Aid
Wandsworth Common
West Dulwich
In-
patients.
5,204
244
2,382
473
214
404
459
138
17
3,194
12,729
12,729
Out-
patients.
38,004
7,669
5,173
6,068
1,161
882
7,045
5,954
6,250
78,206
8,771
4,225
12,432
1,996
2,592
666
5,942
2,249
1,216
712
104
119,111
Total
Expendi-
ture.
?
32,549
3,279
11,606
4,935
1,382
2,912
3,467
1,194
1,019
7,107
69,450
1,881
607
1,829
448
599
289
749
604
39S
156
48
77,058
Income.
Chari-
table.
?
1,133
3,180
6,642
3,739
1,157
792
2,394
1,008
420
6,014
26,479
82
452
366
415
296
65
666
412
76
57
16
Pro-
prietary.
?
26,729
278
4,389
794
2,172
697
353
35,589
34
65
81
103
5
58
29,382 35,935
Patients'
Pa) ments.
?
2,760
143
74
240
192
658
52
4,119
1,812
85
1,271
112
337
215
215
213
99
26
8,504
Total
Income.
?
30,622
3,458
11,174
4,607
1,397
2,964
3,283
1,096
1,167
6,419
66,187
1,928
602
1,718
527
633
280
769
632
347
156
42
73,821
CITY AND EAST CENTRAL DISTRICT.
Comprising the City, St. Luke's, Shoreditch, Finsbury, and Clerkenwell.
Special IVote.?Out of 670 beds provided by the hospitals of this district 383 were constantly occupied during 1887, leaving nearly 300 beds
empty for want of funds. The actual expenditure amounted to ?37,372, and the actua income to ?26,516, leaving a deficiency of nearly ?11,000 as a
debt upon the hospitals for the work done for the poor of the district during 1887, despite the fact that nearly half the available beds were not
occupied throughout the whole of the year. This is a terrible record for the wealthiest rortioa of the wealthiest city in the world.
No. of
Beds
Daily HOSPITALS.
Occu-
pied.
670 1 3S3
Metropolitan ...
Royal Free
Royal Hospital for Diseases of the Chest
North-Eastern, for Children
City of London Lying-in
St. Mark's, for Fistula
Royal London Ophthalmic
City Orthopaedic
Central London Throat and Ear ...
Dispensaries.
City
City of London and East London
Farringdon General ...
Finsbury
Metropolitan
Royal General
Royal Maternity Charity
In-
patients.
1,836
418
654
356
305
2,185
131
340
6,225
6,225
Out-
patients.
26,434
23,541
5,240
13,882
1,204
826
24,547
2,500
5,845
104,019
5,482
6,498
8,105
16,048
5,614
3,681
3,980
153,427
Total
Expendi-
ture.
?
2,753
10,891
4,523
4,892
2,723
2,099
5,919
1,696
1,876
37,372
1,328
879
673
805
774
921
2,724
45,476
Income.
Chari-
table.
?
1,577
4,207
3,010
3,392
417
1,173
2,534
1,481
610
18,401
947
123
257
395
393
496
408
21,420
Pro-
prietary
?
42
1,047
287
243
2,540
818
1,054
67
32
6,130
166
15
99
117
236
947
7,710
Patients'
Payments,
?
52
732
16
1,185
1,985
67 5
372
276
235
95
3,638
Total
Income.
?
1,671
5,254
3,297
4,367
2,973
1,991
3,588
1,548
1,827
26,516
1,113
813
629
770
745
827
1,355
32,768
154 THE HOSPITAL. June 2, 1SS8.
WESTMINSTER DISTRICT.
Comprising Westminster City and Liberties.
Spccial IVote.?Tbebospita's which minister to the needs of the poor of the City of Westminster and its Liberties contain 851 bedf", of whi-h
?63 were daily occupied throughout the year 1887, leaving nearly 25 per cent., or 188 beds unoccupied for want of funds. It will be noticed that the
nomb' r of beds vacant was far less n this than in some other districts, a fact which no doubt accounts for the larger deficiency of ?24,100, which the
managers have to face in consequence. It is worthy of note, that upwards of one-tenth of the whole income of ?52,000 in this d istrict was derived
frcm patients' payments.
No. of Bed.-> TT _ , _
Beds. Daily HOSPITALS.
851 663
Charing Cross
King's College...
Westminster ...
Ventnor, for Consumption ...
Grosvenor, for Women and Children
Gordon, for Fistula ...
Royal Westminster Ophthalmic
Royal Orthopaedic ... ...
Hospital for Diseases of the Throat
Dispensaries.
Public
SS. George and James
St. George, Hanover Square
Western
Westminster General
In-
patients.
1,686
1,811
2,580
666
95
95
356
132
312
7,733
7,733
Out-
patients
20,306
17,248
20,912
2,128
406
7,524
1,129
5,218
74,471
3,445
3,685
2,750
3,023
5,614
92,988
Total
Ex; endi-
ture.
?
12,912
25,476
16,749
13,387
932
937
1,779
2,094
2,614
76,880
704
711
662
967
497
80,421
Income.
Chari-
table.
?
6,190
11,093
9,179
4,784
531
427
1,419
1,612
721
35,956
430
418
522
847
483
38,656
Pro- J Patients'
prietary. j Payments,
?
3,781
1,953
3,049
1,602
11
"296
341
61
11,094
171
22
314
140
11,741
?
117
2,520
410
362
61
120
2,022
5,612
160
217
46
6,035
Total
Income.
ST. MARYLEBONE AND WEST CENTRAL DISTRICT.
Comprising St. Marylebone, St. John's Wood, Bloomsbury, Holborn, etc.
Special Note.? This district contains 1,245 hospital beds, of which 837, or something like 66 per cent., were constantly oocupi d throughout
1887. The total expenditure amounted to ?83,700, and the total income to ?68,500, leaving a deficiency of ?15,200, which would have been increased
to ?65,000 at least had the whole of the available bads been filled with patients throughout the year.
No. of
No. of Beds I jn_
Beds. Dai'y HOSPITALS. ! patients.
Occu-
pied.
1,245
French ...
Italian ...
SS. John and Elizabeth
The Middlesex
Alexandra, for Children
Home for Incurable Children
Hospital for Sick Children .
British Lying-in
Queen Charlotte's Lying-in.
New Hospital for Women ...
National, for the Paralysed, etc.
Central London Ophthalmic
Samaritan Free
Western Ophthalmic... !..
National Orthopcedic
St. John's, for Diseases of the Skin
London Homoeopathic
Dental
25 10 Establishment for Gentlewomen
Dispensaries.
Margaret Street Infirmary .
Portland Town
Portobello Road
St. John's Wood Provident
St. Marylebone General
Western General
422
197
92
2,413
144
40
1,494
150
962
256
613
197
491
54
134
130
711
112
8,612
8,612
Out-
patients.
3,315
2,279
27^714
152
15 j 647
600
1,196
3,348
3,229
8,427
4,642
1,926
701
4,350
8,824
32,235
118,585
1,448
2,605
103
1,304
4,336
19,514
147,895
Total
Expendi-
ture.
?
2,787
794
1,451
26,348
3,451
1,368
10,862
1,700
3,623
1,843
9,070
1,073
5,105
682
1,058
3,530
5,031
1,450
2,511
83,737
424
143
51
479
796
1,484
87,114
Income.
Chari-
table.
?
2,810
927
847
9,591
1,693
694
8,944
600
2,208
1,003
4,970
537
4,175
419
638
2,412
4,306
1,775
971
49,520
405
134
46
294
364
1,410
52,173
Pro-
prietary.
?
265
21
641
5,478
103
15
734
996
322
266
1,404
70
25
103
39
2,277
183
113
13,055
154
13
130
49
Patients'
Payments
26
565
385
"'26
84
444
1,362
213
167
579
597
361
240
898
5,941
13,401
25
274
316
6,564
June 2, 1^87 THE HOSPITAL.  15 5
KENSINGTON AND WEST DISTRICT.
Comprising Kensington, Paddington, Bayswater, Kilburn, Chelsea, Brompton, Fulham, Hammersmith, Chiswick, Brentford,
Acton, Ealing, etc.
Specinl Note.?The 16 hospitals Fitna'ed in 1his disliict. contain 1,' 96 beds, of which 1,261, or something- like 75 per cent., were constantly
occupied throughout 1887. The total expenditure amounted to ?111,400, and the total income to ?95,100, leaving a deficiency of ?16,300. Large as
this deficiency is, it is smaller than that of any other district, but it would have been increased to something like ?15,000 had all the available beds
been constantly occupied throughout the yeir.
No. of
Beds _T
Daily HOSPITALS.
Occu-
pied. i
St. George's ...
St. Mary's
West London
Hospital for Consumption
Belgrave, for Children
Cheyne, for Incurable Children
Paddington Green, for Children
Victoria, for Children
Chelsea, for Women...
Hospital for Women...
Cancer
National, for Diseases of Heart
Female Lock
Male Lock
National Dental
St. Mary Magdalene's Home
Dispensaries.
Brompton Provident
Chelsea, Brompton, etc.
Kensington
Kilburn, MaidaVale
Kilburn Provident ...
Notting Hill Provident
Paddington Provident
Royal Pimlico Provident
SS. Paul and Barnabas
Westbourne Provident
In-
patients.
1.261
3,258
3,315
1,223
1,215
135
52
335
7G2
438
620
703
85
599
187
56
12,983
Out-
patients.
22,410
26,637
16,522
13,623
2,343
10,776
10,869
3.450
5,321
1,012
2,560
2^584
23,965
142,072
813
4,624
5,058
3,782
3,480
1,580
7,559
11,853
2,566
3,349
12,983 1186,736
Total
Expendi-
ture
?
26,961
19,691
5,386
22,173
1,405
1,399
2,050
4,121
3,402
7,877
8,616
2,036
3,460
1,186
821
857
111,441
374
619
838
502
1,067
259
597
893
497
391
117,478
Income.
Chari-
table.
?
11,843
12,23S
5,812
14,109
2,225
1,414
3,800
3,757
2,835
4,598
5,423
1,658
2,621
298
305
434
73,370
162
343
776
449
96
148
217
326
362
105
76,354
Pro-
prietary
?
8,016
1,391
81
4,197
12
195
139
653
53
172
916
84
42
15,951
114
40
36
94
20
27
13
16,310
Patients'
Payments.
198
127
298
781
1,659
284
954
700
436
395
5,832
223
878
81
390
542
126
288
Total
Income.
?
19,859
13,629
5,893
18,306
2,237
1,807
4,066
4,708
3,669
6,429
6,339
2,026
3,575
998
783
829
95,153
392
457
816
485
1,068
249
634
881
488
401
8,360
101,024
ISLINGTON AND NORTH-WEST DISTRICT.
Comprising Islington, Holloway, Highbury, Hampstead, St. Pancras, Tottenham, etc.
Special Note.?This is one of the largest and most populous districts of the metropolis, but it only ontains 761 beds, of which 563 were
constantly occupied. The number of vacant beds was therefore relatively fewer than iu any other district, a* mar well be imagined, owing to the
density of the population, and the comparatively few hospital beds available. The total expenditure am mat-id to ?57,10), and the totil income to
?,">4,000, being a deficiency of upwards of ?3,000, which would have been increased to ?17,000 at least hai all the available be is been occupied.
No. of
Beds
Daily
Occu-
pied.
Hospitals.
29 Great Northern Central
36 North-West London
52 Tottenham
179 University College ...
37 North London Consumption
25 St. Saviour's, for Cancer
100 London Fever
17 Hospital for Epilepsy, etc.
59 London Temperance...
9 Hampstead Home Hospital
20 Invalid Asylum
563
Dispensaries.
Child's Hill
Hampstead Provident
Hollo way and North Islington
Islington
St. Pancras and Northern ...
Stamford Hill, etc. ...
563
In-
patient?
326
520
620
2,964
320
119
982
116
674
116
211
6,968
6,968
Out-
pitients.
10,000
11,160
5,384
44,382
2,900
349
677
2,595
77,447
578
9,402
4,569
15,882
2,375
5,242
115,495
Total
Expendi-
ture.
?
3,509
3,600
3,282
20,305
4,304
3,309
9,178
2,237
5,037
1,367
1,013
57,141
266
883
845
1,930
521
606
62,192
Income.
Chari-
table.
?
2,102
3,225
2,209
12,796
5,471
365
7,282
1,334
4,424
428
627
40,263
54
311
521
603
325
491
42,56S
Pro-
prietary.
?
272
40
555
4,328
80
78
1,045
270
76
73
6,817
11
14
"794
106
101
7,843
Patients'
Payments,
125
332
55
54
870
3,873
526
265
468
231
6,799
207
556
435
530
74
8,601
Total
Income.
?
2,374
3,390
3,096
17,179
5,605
1,313
12,200
1,860
4,959
972
931
53,879
272
881
956
1,927
505
592
59,012
156
THE HOSPITAL. June 2, it
STRATFORD AND EAST-END DISTRICT.
Comprising Bethnal Green, Tower Hamlets, West Ham, Whitechapel, Hackney, Stepney, Limeliouse, Poplar,
and the East.
Special IVole,?This district contains 1,312 beds, of which 1,037 were constantly occupied during the year 18S7, leaving 275 beds empty for
want of funds. The total expenditure amounted to ?87,000, and the total income to ?71,600, so that the deficiency amounted to ?15,4.0 in round
numbers. Tliis deficiency would hive been increased to ?32,800 had the available beds been occupied throughout tha year.
No. of
Beds.
125
772
50
164
102
7
92
1,312
1,312
No. of
Beds
Daily
Occu-
pied
110
644
35
107
74
3
64
1,037
1,037
Hospitals.
German...
London
Poplar
City of London for Diseases of the Chest
East London for Children ...
Mothers' Lying-in Home
Mrs. Gladstone's Home
Dispensaries
Eastern...
London...
Queen Adelaide's
Tower Hamlets
West Ham, Stratford, etc.
In-
patients.
1,264
8,260
678
946
1,018
84
1,118
13,368
13,368
Out-
patients.
20,985
95,760
9,762
15,810
14,994
12
157,323
5,334
3,032
4,301
3,530
16,079
189,599
Total
Expendi-
ture.
?
9,269
55,642
3,090
9,812
6,804
894
1,508
87,019
623
419
428
737
837
90,063
Income.
Chari-
table.
?
6,143
24,741
3,204
6,454
5,939
665
986
48,132
272
212
611
744
924
50,895
Pro- Patients'
prietary. Payments.
? ?
2,224 235
19,245 104
346
487
506
362
23,164 339
267 47
269
130
28 128
134
23,992 514
SUMMARY.
It will be seen from the following Summary that the Hospitals and Medical Charities of London during the twelve months
ending 31st December, 1887, relieved nearly 1,260,000 patients, at a cost of ?631,800. The ordinary income was ?560,500,
leaving a deficiency on the year's working of upwards of ?90,000.
^Bedsf Daily HOSPITALS AXD DISPENSARIES.
Newington and South District
City and East Central do.
Westminster do.
St. Marylebone and West Central do.
Kensington and West do.
Islington and North-West do. ...
Stratford and East-end do.
St. Thomas's Hospital
St. Bartholomew's ...
In-
patients.
12,729
6,225
7,733
8,612
12,983
6,968
13,368
68,618
4,643
6,000
79,261
Oat-
patients.
119,111
153,427
92,988
147,895
186,736
115,495
189,599
1,005,251
25,000
150,000
1,180,251
Total
Expendi-
ture.
?
77,058
45,476
80,421
87,114
117,478
62,192
90,063
559,802
32,000
60,000
651,802
Income.
Chari- Pro-
table. j prietary. Payments
?
29,382
21,420
38,656
52,173
76,354
42,568
50,895
?
35,935
7,710
11,741
13,401
16,310
7,843
23,992
311,448 116,932
Patients'
?
8,504
3,638
6,035
6,564
8,360
8,601
514
42,216

				

